# Supplementary material for: Optimization of Extraction Process, Structure Characterization, and Antioxidant Activity of Polysaccharides from Different Parts of Camellia oleifera Abel
Source: Foods. 2022 Oct 12;11(20):3185. doi: 10.3390/foods11203185 (PMC9602086; doi:10.3390/foods11203185)
Supplement: Supplementary file 1 [file foods-11-03185-s001.zip › foods-1923577-supplementary.pdf]

Supplementary Materials

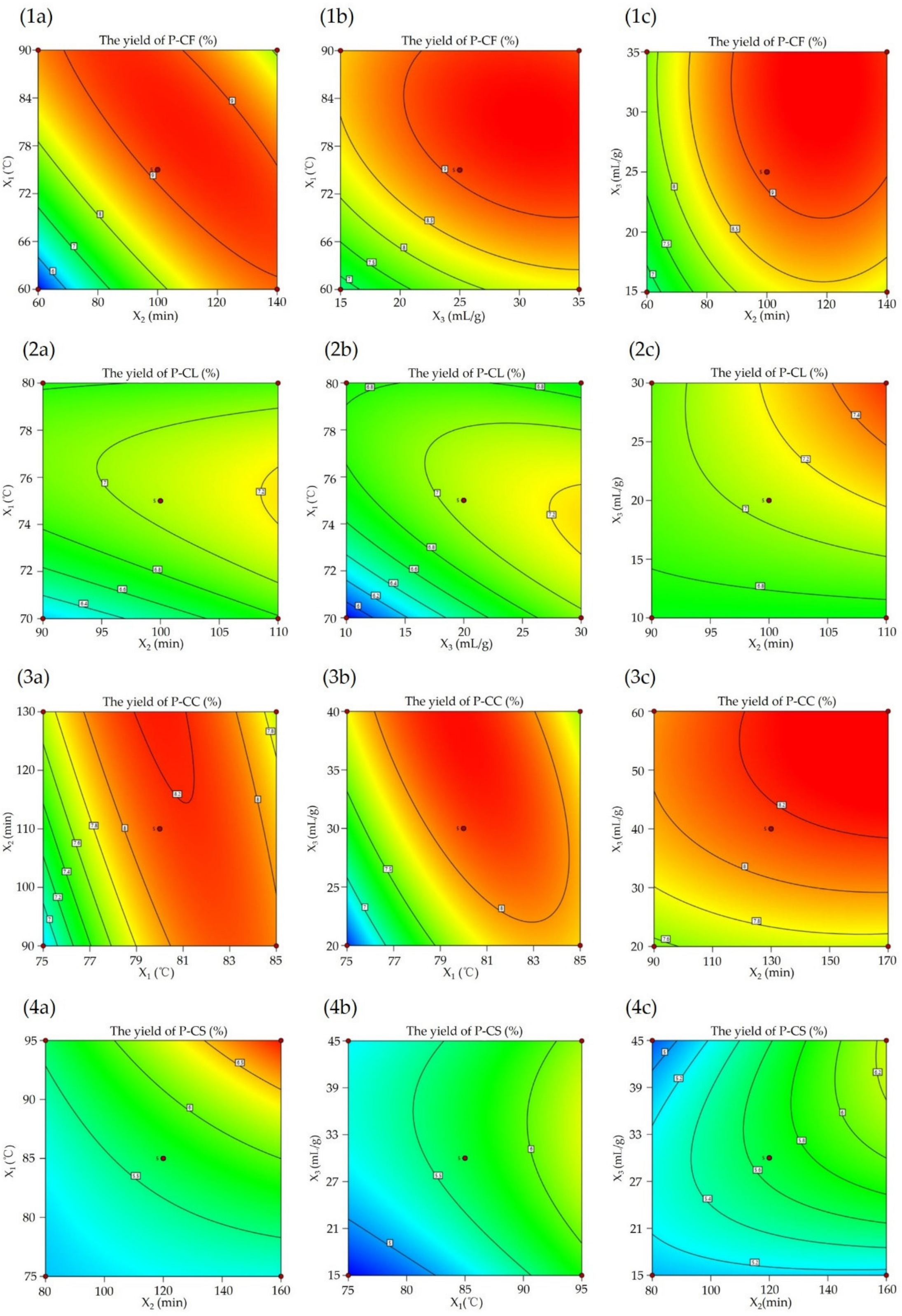

Figure S1. Contour plots of P-CF, P-CL, P-CC and P-CS. Contour plots of the interactions between (a) extraction temperature and extraction time, (b) extraction temperature and liquid-solid ratio, (c) extraction time and liquid-solid ratio (for (1) P-CF, (2) P-CL, (3) P-CC, (4) P-CS). X1, X2 and X3 represent the extraction temperature, extraction time and Liquid-Solid Ratio, respectively.
